# Dental anomalies in an orthodontic patient population with maxillary lateral incisor agenesis

**DOI:** 10.1590/2177-6709.21.6.098-102.oar

**Published:** 2016

**Authors:** Mehmet Citak, Elif Bahar Cakici, Yasin Atakan Benkli, Fatih Cakici, Bircan Bektas, Suleyman Kutalmış Buyuk

**Affiliations:** 1 Research Assistant, Department of Endodontics, Faculty of Dentistry, Ordu University, Ordu, Turkey.; 2 Assistant Professor, Department of Endodontics, Faculty of Dentistry, Ordu University, Ordu, Turkey.; 3 Assistant Professor, Department of Orthodontics, Faculty of Dentistry, Ordu University, Ordu, Turkey.; 4 Research Assistant, Department of Orthodontics, Faculty of Dentistry, Ordu University, Ordu, Turkey.

**Keywords:** Dental anomalies, Hypodontia, Panoramic radiograph

## Abstract

**Introduction:**

The purpose of this study was to evaluate the prevalence of dental anomalies in a subpopulation of orthodontic patients with agenesis of maxillary lateral incisors (MLI).

**Methods:**

The material of the present study included the records of the 1964 orthodontic patients. Panoramic radiographs and dental casts were used to analyze other associated eight dental anomalies, including agenesis of other teeth, *dens invaginatus*, *dens evaginatus*, peg shaped MLI, taurodontism, pulp stone, root dilaceration and maxillary canine impaction.

**Results:**

Out of the 1964 patients examined, 90 were found to have agenesis of MLI, representing a prevalence of 4.6%. The most commonly found associated-anomalies were agenesis of other teeth (23.3%), peg-shaped MLIs (15.6%), taurodontism (42.2%), and dilacerated teeth (18.9%).

**Conclusion:**

Permanent tooth agenesis, taurodontism, peg-shaped maxillary lateral incisor, and root dilacerations are frequently associated with maxillary lateral incisor agenesis.

## RESUMO

**Objetivo:**

o objetivo do presente estudo foi avaliar a prevalência de anomalias dentárias em uma subpopulação de pacientes ortodônticos com agenesia de incisivos laterais superiores (ILS).

**Material e Métodos:**

o material do presente estudo incluiu os registros de 1964 pacientes ortodônticos. Radiografias panorâmicas e modelos de estudo foram usados para analisar outras anomalias dentárias associadas, incluindo a agenesia de outros dentes, *dens invaginatus*, *dens evaginatus*, ILS conoides, taurodontismo, calcificação pulpar, dilaceração radicular e impacção do canino superior.

**Resultados:**

dos 1964 pacientes examinados, constatou-se que 90 tinham agenesia do ILS, o que representa uma prevalência de 4,6%. As anomalias associadas mais comumente encontradas foram a agenesia de outros dentes (23,3%), ILS conoides (15,6%), taurodontismo (42,2%) e dentes com dilaceração (18,9%).

**Conclusão:**

a agenesia de dentes permanentes, o taurodontismo, incisivos laterais superiores conoides e dilaceração radicular estão frequentemente associados à agenesia de incisivos laterais superiores.

## INTRODUCTION

Dental anomalies are typically caused by either genetic or environmental stimuli.^1^ ^,^ ^2^ Mutations in AXIN2, PAX9 and MSX1 have been determined in families with dental agenesis.^3^ ^,^ ^4^ The most frequently observed dental agenesis in children is defined as the absence of one or more primary/permanent teeth.^5^ Data for congenital tooth agenesis prevalence vary between 0.3 and 11.3%^6^ ^,^ ^7^ for both males and females. However, the prevalence of congenital tooth agenesis was shown to be higher in females than in males, in some reports.^5^ ^,^ ^8^ ^,^ ^9^

After third molars, maxillary lateral incisors (MLI) are the teeth that are the most frequently missing.^5^ ^,^ ^10^ Agenesis of MLI has been documented for its higher prevalence than of other permanent teeth.^11^ A correlation between MLI agenesis and palatally displaced canines,^12^ tooth transpositions,^13^ and premolar rotations^14^ has also been reported. However, reports on the prevalence of dental anomalies in a large MLI agenesis patient cohort have not been determined. The purpose of the current study was to investigate the prevalence of MLI agenesis and other dental anomalies in an orthodontic subpopulation in Turkey. 

## MATERIAL AND METHODS

Panoramic radiographs of 1964 patients (1174 females, 790 males) of the Department of Orthodontics of Ordu University, Turkey, between January 2013 and September 2015, were retrospectively analyzed. Patients, aged 12 to 25 years, with unilateral or bilateral agenesis of MLI and panoramic radiograph were included in the study. Patients with incomplete records, permanent tooth extraction, and/or poor-quality panoramic radiographs were excluded from the study. Care was used to ensure that all radiographs were taken by the same technician operating the same panoramic roentgen unit device (Kodak Cephalostat, Rochester NY, USA). In order to eliminate inter-examiner differences, all records were examined by one observer. All radiographs were evaluated by an orthodontist with more than 15 years of experience. 

**Figure 1:**
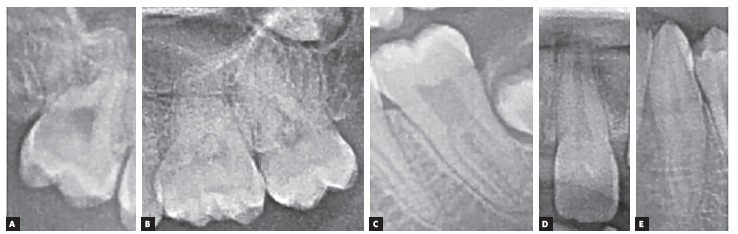
A) Root dilacerations. B) Pulp stone. C) Taurodontism. D) *Dens evaginatus*. E) *Dens invaginatus*.

The following anomalies were determined in this study (Fig 1):

1) Agenesis: congenital developmental loss of one or more permanent teeth.
2) *Dens invaginatus*: caused by the invagination of enamel into the dental papilla before the mineralization phase.^15^
3) *Dens evaginatus*: malformation characterized by an accessory cusp, composed of normal enamel and dentine, with or without pulp tissue.^16^
4) Microdontia (peg-shaped teeth): teeth that are substantially smaller than the average normal size. Microdontia also refers to a tooth that does not fill its space in the dental arch or appears small due to absence of expected shape.^17^
5) Taurodontism: vertically extended, extremely oversized pulp cavities that are apically displaced at the pulpal floor.^18^
6) Pulp stone: calcified masses on the pulp of healthy, diseased, and even unerupted teeth freely attached or embedded into the coronal rather than the root portion of the pulp organ.^19^
7) Dilaceration: deviation in the linear relationship of a crown of a tooth to its root.^20^
8) Impaction: a tooth that is predicted to remain unerupted because of a physical barrier or deflection along its eruption path.^21^

### Statistical analysis

Statistics were calculated with SPSS 15.0 statistical software (SPSS Inc, Chicago, IL, USA). Anomaly prevalence was measured with respect to sex, side and dental location. Chi-square analysis and MLI prevalence were compared to previously published reports from the Turkish population.^22^ ^-^ ^28^ A p-value of less than 0.05 was considered significant.

To examine errors associated with digitizing and measurements, 10% of panoramic images were selected randomly and all dental anomalies were evaluated by the same author four weeks after the first examination. Kappa coefficients were used to calculate the reliability of each dental anomaly determination from the two evaluation periods. 

## RESULTS

Kappa score of each dental anomaly was 1.00. This score indicated good agreement with the first and second evaluations and was observed for each dental anomaly. Out of the 1964 subjects (1174 females, 790 males) evaluated, 90 (62 females, 28 males) were determined to have MLI agenesis (prevalence = 4.58%; being 5.3% for females and 3.5% males. Difference between males and females was statistically not significant [X^2^ = 3.26; *p*= 0.071]). Bilateral MLI agenesis was found in 62 subjects (68.9%) and unilateral agenesis in 28 patients (31.1%). 

The investigated dental anomalies in MLI agenesis patients were: *dens invaginatus*, *dens evaginatus*, peg-shaped MLI, taurodontism, pulp stone, root dilacerations, impaction of maxillary canine, and missing teeth other than third molars (Table 1). The prevalence of MLI agenesis-associated dental-anomalies was referenced to previous work, for consistency (Table 2). The prevalence of agenesis of other teeth (*p*< 0.001), peg-shaped MLIs (*p*< 0.001), taurodontism (*p*< 0.001), and dilacerated teeth (*p*< 0.01) were greater in our sample compared to the other studies. No statistical significant difference was shown for the prevalence of *dens invaginatus* (*p*= 0.888), *dens evaginatus* (*p*= 0.123), pulp stone (*p*= 0.666) and impaction of maxillary canines (*p*= 0.477).

**Table 1:** Distribution of MLI agenesis-associated dental-anomalies.

Dental anomaly	Male / Female	Unilateral / Bilateral	Total (%)
Agenesis of other teeth	5 / 16	7 / 14	21 (23.3)
*Dens invaginatus*	1 / 0	1 / 0	1 (1.1)
*Dens evaginatus*	1 / 1	1 / 1	2 (2.2)
Peg shaped MLI	7 / 7	14 / 0	14 (15.6)
Taurodontism	9 / 29	1 / 37	38 (42.2)
Pulp stone	0 / 9	5 / 4	9 (10)
Dilaceration	4 / 13	5 / 12	17 (18.9)
Impaction of maxillary canine	2 / 3	3 / 2	5 (5.6)

**Table 2:** Comparison of the frequencies of dental anomalies subjects with maxillary lateral incisor agenesis and previous studies.

Dental anomaly	Present study			Reference studies			P*
	n	%	n	Total	%	Literature	
Agenesis of other teeth	21	23.3	144	3165	5.0	Kazanci et al.^22^	< 0.001
*Dens invaginatus*	1	1.1	13	1012	1.3	Cakici et al.^23^	0.888
*Dens evaginatus*	2	2.2	56	900	6.2	Uslu et al.^24^	0.123
Peg shaped MLI	14	15.6	46	3043	1.5	Altug-Atac, Erdem^25^	< 0.001
Taurodontism	38	42.2	9	900	1.0	Uslu et al.^24^	< 0.001
Pulp stone	9	10	60	519	11.6	Gulsahi et al.^26^	0.666
Dilaceration	17	18.9	214	2251	9.5	Miloglu et al.^27^	0.003
Impaction of maxillary canine	5	5.6	488	12000	4.1	Gunduz, Celenk^28^	0.477

n = number of subjects, MLI; Maxillary lateral incisor, * *p* indicates results of chi-square test.

## DISCUSSION

The prevalence of dental anomalies is variable among different populations. The aim of the present study was to determine the prevalence of MLI agenesis-associated dental anomalies in orthodontic Turkish patients. We found that about 4.58% of patients had one or both maxillary incisors missing. These results are consistent with that reported by others, which ranges from 0.3% and 11.3%.^9^ ^,^ ^10^ ^,^ ^25^ The prevalence of MLI agenesis varies considerably between studies.^7^ ^,^ ^29^ ^-^ ^32^ Horowitz^33^ showed a prevalence of 1.11% in an adolescent population (n = 1000; ages ranging from 7 to 16 years). Celikoglu et al^7^ reported a prevalence of 2.4% from 3872 East Anatolian adolescent patients in Turkey. The differences may be related to sample size or selection, but may be different due to regional ethnic population, genetic variability, and/or environmental factors. 

There was no statistically significant correlation between sex and MLI agenesis. Interestingly, we had more female subjects in our cohort. Some reports have shown insignificant differences,^34^ ^,^ ^35^ while others have determined significant sex-related changes.^36^ ^,^ ^37^

Previous studies^24^ ^,^ ^38^ ^-^ ^41^ have reported that tooth agenesis can be associated with other dental malformations, such as taurodontism, transposition, microdontia, ectopic eruption, supernumerary tooth or peg-shaped MLI. Most of the papers^8^ ^,^ ^42^ ^,^ ^43^ published on MLI agenesis demonstrated a reduction in crown size or a peg-shaped form of the contralateral MLI. MLI agenesis was detected more commonly in females than males.

When we compared the prevalence rates of MLI agenesis-associated dental anomalies and reference values,^22^ ^-^ ^28^ it was determined that the prevalence rates were significantly augmented for taurodontism, agenesis of other teeth, and peg-shaped MLIs. There have been only two studies that have compared their work with^7^ ^,^ ^38^ reference values. However, taurodontism, pulp stone, *dens invaginatus*, *dens evaginatus*, and impaction of the maxillary canine were not assessed in those studies. In this respect, our study is the first one to show the different associated dental anomalies between subjects with MLI agenesis. 

The most common MLI agenesis-associated dental anomaly was taurodontism; in our study, with a prevalence of 42.2%. Uslu et al^24^ showed 1% of taurodontism prevalence in 900 orthodontic patients. Discrepancy in the results may be related to the location variations, and our taurodontism detection method. The difference might arise from racial differences or differences in diagnostic criteria.

Out of the 28 patients who had unilateral absence, 14 (50.0%) were found to have a peg-shaped lateral incisor on the other side. Altug-Atac and Erdem^25^ reported that 1.51% of patients had peg-shaped MLI in an orthodontic patient population. However, Albashaireh and Khaider^42^ demonstrated that peg-shaped and reduced size maxillary lateral incisors were found in 2.3% and 2.9% of patients, respectively. Similar to the prevalence of our results, Albashaireh and Khader^42^ showed 50% microdontia or peg-shaped MLIs on the other side in individuals with unilateral MLI agenesis. In this study, the prevalence of other teeth agenesis (23.3%) was very high, compared to the reports by Celikoglu et al.^7^ The higher rate reported may be attributed to orthodontic malocclusions, which supports the findings by Garib et al.^38^

The prevalence of root dilacerations in our study (18.9%) was greater than that reported of a reference study from the general population (9.5%).^27^ Diagnosing dilacerations is mostly imperative for root canal treatment, tooth extraction, and Orthodontics.^34^ The other associated anomalies (*dens invaginatus, dens evaginatus*, pulp stone and impaction of maxillary canine prevalence) were similarly found with reference studies.^23^ ^,^ ^24^ ^,^ ^26^ ^,^ ^28^

Associations between tooth anomalies are clinically relevant, and early diagnosis may be helpful to reduce risk.^44^ Therefore, diagnosis and treatment options should be precisely made. We found a higher prevalence of associated dental anomalies in MLI agenesis patients in Turkish orthodontic population. However, the orthodontic literature shows different prevalence rates of dental anomalies from the general population.^7^ ^,^ ^9^ ^,^ ^22^ ^,^ ^25^ ^,^ ^34^ This is mostly likely due to the greater variability of racial factors, environmental stimuli and genetics.

## CONCLUSIONS

The increased prevalence of MLI agenesis-associated dental anomalies was validated by previous reports from another orthodontic patient population. There was a significant correlation between MLI agenesis and the agenesis of other permanent teeth. In addition, increased agenesis of other teeth, taurodontism, peg-shaped maxillary lateral incisor and root dilacerations were also statistically significant. These associations can be most likely explained by genetic or environmental factors that may contribute to these dental anomalies.
